# Eicosanoid lipidome activation in post-mortem brain tissues of individuals with *APOE4* and Alzheimer’s dementia

**DOI:** 10.1186/s13195-022-01084-7

**Published:** 2022-10-11

**Authors:** Brandon Ebright, Isaac Assante, Roy A. Poblete, Shaowei Wang, Marlon V. Duro, David A. Bennett, Zoe Arvanitakis, Stan G. Louie, Hussein N. Yassine

**Affiliations:** 1 School of Pharmacy, University of Southern California, Los Angeles, USA; 2 Keck School of Medicine, University of Southern California, Los Angeles, USA; 3 Rush Alzheimer’s Disease Center, Rush University Medical Center, Chicago, IL USA

**Keywords:** Alzheimer’s disease, ApoE, Lipidomics, Inflammation

## Abstract

**Background:**

Chronic neuroinflammation is one of the hallmarks of late-onset Alzheimer’s disease (AD) dementia pathogenesis. Carrying the apolipoprotein ε4 (*APOE4*) allele has been associated with an accentuated response to brain inflammation and increases the risk of AD dementia progression. Among inflammation signaling pathways, aberrant eicosanoid activation plays a prominent role in neurodegeneration.

**Methods:**

Using brains from the Religious Order Study (ROS), this study compared measures of brain eicosanoid lipidome in older persons with AD dementia to age-matched controls with no cognitive impairment (NCI), stratified by *APOE* genotype.

**Results:**

Lipidomic analysis of the dorsolateral prefrontal cortex demonstrated lower levels of omega-3 fatty acids eicosapentaenoic acid (EPA), docosapentaenoic acid (DPA), and DHA-derived neuroprotectin D1 (NPD-1) in persons with AD dementia, all of which associated with lower measures of cognitive function. A significant interaction was observed between carrying the *APOE4* allele and higher levels of both pro-inflammatory lipids and pro-resolving eicosanoid lipids on measures of cognitive performance and on neuritic plaque burden. Furthermore, analysis of lipid metabolism pathways implicated activation of calcium-dependent phospholipase A2 (cPLA2), 5-lipoxygenase (5-LOX), and soluble epoxide hydrolase (sEH) enzymes.

**Conclusion:**

These findings implicate activation of the eicosanoid lipidome in the chronic unresolved state of inflammation in AD dementia, which is increased in carriers of the *APOE4* allele, and identify potential therapeutic targets for resolving this chronic inflammatory state.

**Supplementary Information:**

The online version contains supplementary material available at 10.1186/s13195-022-01084-7.

## Introduction

It is estimated that approximately 50 million individuals suffer from dementia worldwide, where the vast majority of cases are attributed to Alzheimer’s disease (AD) [1]. Without the introduction of effective therapies, the global prevalence of AD is expected to quadruple by 2050 [2]. Amyloid beta (Aβ) senile plaques and neurofibrillary tangles (NFTs) are associated with neuronal injury and cognitive decline, and for decades, most AD research aimed to understand how their deposition may lead to dementia onset and progression. Unfortunately, attempts to block their molecular pathogenesis with therapeutics development have not led to clinically effective strategies against AD.

In addition to Aβ and NFT aggregates, chronic inflammation represents an important hallmark of AD pathology. In the brain, chronic inflammation is largely driven by the dysregulation of pro-inflammatory cytokines released from activated microglia and astrocytes [3, 4]. Lipid peroxidation has been closely linked to sustaining chronic inflammation where oxygen radicals mediate the formation of reactive lipid intermediates that play a role in neurodegeneration [5, 6]. In large observational cohorts, systemic inflammatory markers like C-reactive protein (CRP) were associated with both brain amyloid deposition and cognitive decline later in life [7, 8].

**Fig. 1:**
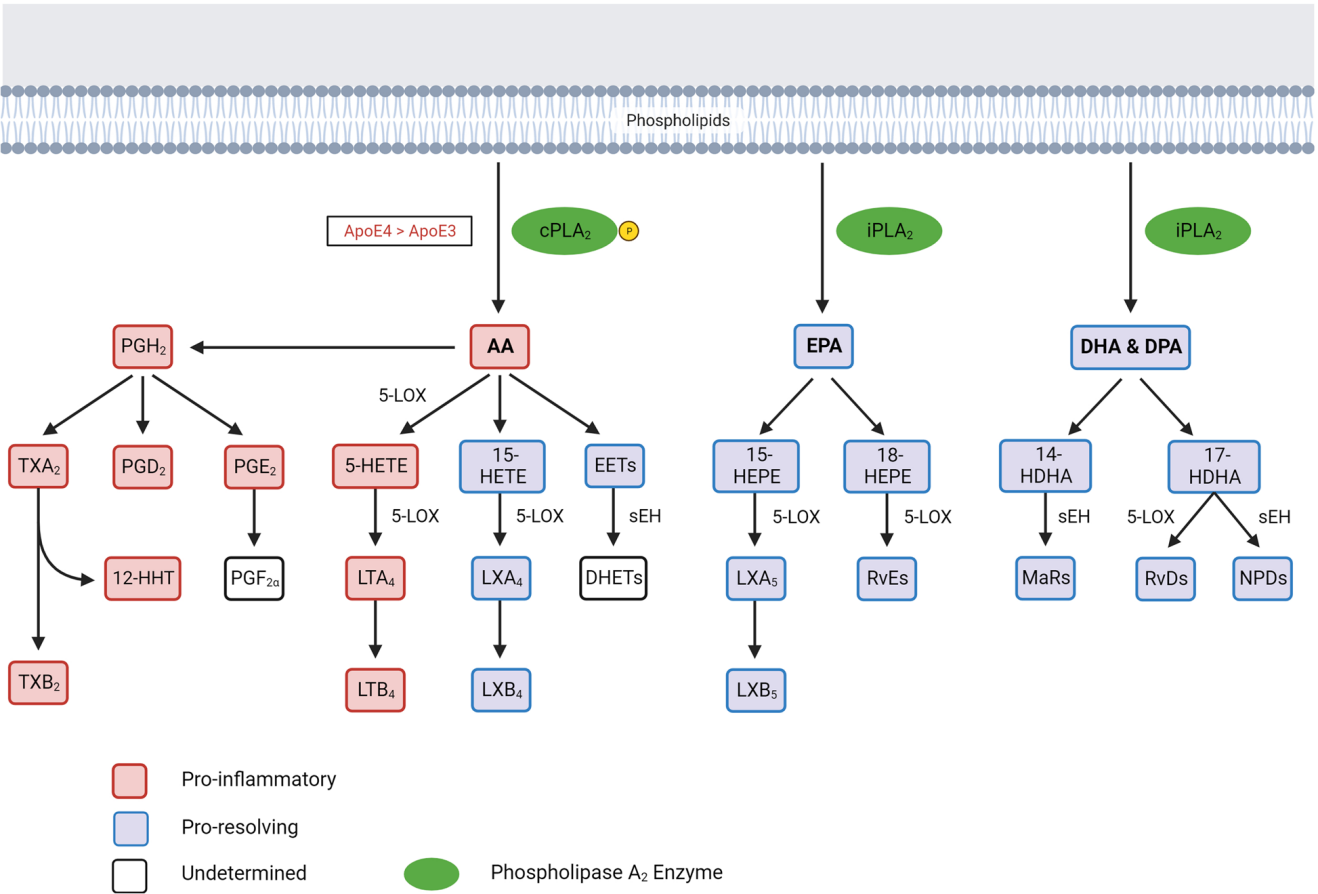
PUFAs, eicosanoids, and specialized pro-resolving lipid mediators (SPMs) and their regulation by phospholipase A2, 5-LOX, and sEH enzymes. ApoE4 sustains cPLA2 activation more than ApoE3. Red squares represent inflammatory components, while blue squares represent pro-resolving mediators. White squares are analytes where biological activity has not been clearly ascertained. Abbreviations: cPLA2, calcium-dependent phospholipase A2; iPLA2, calcium-independent phospholipase A2; AA, arachidonic acid; EPA, eicosapentaenoic acid; DHA, docosahexaenoic acid; DPA, docosapentaenoic acid; PGs, prostaglandins; TXs, thromboxanes; 12-HHT, 12-hydroxyheptadecatrienoic acid; HETEs, hydroxyeicosatetraenoic acids; HEPEs, hydroxyeicosapentaenoic acids; HDHAs, hydroxydocosahexaenoic acids; LTs, leukotrienes; LXs, lipoxins; EETs, epoxyeicosatrienoic acids; DHETs, dihydroxyeicosatrienoic acids; RvEs, E-series resolvins, MaRs, maresins; RvDs, D-series resolvins; NPDs, neuroprotectins; 5-LOX, 5-lipoxygenase; sEH, soluble epoxide hydrolase

Dysregulated lipid metabolism has also been tied to neuroinflammation. In particular, individuals who carry the Apolipoprotein E4 allele (*APOE4*) have an increased risk for AD dementia [9–13]. ApoE is a polymorphic apolipoprotein that functions as a lipid and cholesterol transporter. In addition, ApoE is also involved in various cellular functions such as neuronal signaling, neuroinflammation, glucose metabolism, and transporting lipids into brain cells, where dysfunction or dysregulation in lipid transport has been implicated in AD pathogenesis. The molecular mechanism by which this genotype affects neuroinflammation has not been fully delineated; however, we recently identified higher cytosolic phospholipase A_2_ (cPLA2) activation in *APOE4* cellular and animal models as well as human brain tissues [14]. cPLA2 activation liberates arachidonic acid (AA) from phospholipids of cellular membranes to promote brain inflammation through generating eicosanoid lipids (Fig. 1). Among inflammation signaling pathways, eicosanoids and specialized pro-resolving lipid mediators (SPMs) have important roles in maintaining homeostasis in the presence of pro-inflammatory or otherwise noxious stimuli. Dysregulation of SPM generation has been demonstrated in human brains with AD and rodent models [14–16].

The aim of this investigation was to define the brain eicosanoid and SPM lipidome as a function of dementia, cognitive function, and *APOE4* genotype, in human postmortem tissue from persons with different *APOE4* statuses. We hypothesize that AD dementia is associated with eicosanoid-driven inflammation, as manifested by elevated measures of lipid neuroinflammation markers and lower measures of SPMs, and that this molecular phenotype is increased in *APOE4* carriers with AD dementia. Furthermore, pathways analysis will be explored to identify the key regulating enzymes that regulate brain inflammation as potential targets of therapy.

## Results

### Dysregulation of omega-3 and omega-6 free fatty acid content in the AD dementia brain

To characterize the lipidomic profile of brains with AD, postmortem cortical tissues were compared to age-matched controls with no cognitive impairment (NCI). NCI is defined as persons with no perceived decline in cognitive abilities. The demographic characteristics of tested brain samples are summarized in Table 1. When separated by *APOE* genotype and clinical diagnosis, age and education level were different between *APOE3/3* NCI and *APOE3/3* AD dementia groups according to one-way ANOVA tests. Age, and Braak stage, were also different between *APOE3/4* NCI and *APOE3/4* AD dementia groups, as well as for comparisons between *APOE3/3* NCI and *APOE3/4* AD groups. The proportion of males and females was not significantly different between groups by chi-squared test.

**Table 1:** Demographic characteristics of human brain samples

Brain region and source	Dorsolateral prefrontal cortex (ROSMAP, RADC)			
Clinical diagnosis	NCI	NCI	AD dementia	AD dementia
Genotype	E3/E3	E3/E4	E3/E3	E3/E4
Sample size, *n*	12	9	12	9
Age at death, years ± SD^a^	85 ± 6	87 ± 5	92 ± 6	94 ± 5
Sex, female (%)^b^	6 (50)	5 (56)	5 (42)	6 (67)
Education, years ± SD^c^	21 ± 3	19 ± 2	17 ± 3	17 ± 3
Global cognitive function ± SD^d^	0.3 ± 0.3	0.4 ± 0.4	−1.7 ± 1.0	−2.8 ± 0.8
Neuritic plaque burden ± SD^e^	0.5 ± 0.7	0.3 ± 0.5	0.9 ± 0.6	1.9 ± 0.6
Overall amyloid level ± SD^f^	2.8 ± 3.6	3.2 ± 4.6	5.2 ± 4.7	7.7 ± 4.9
Braak stage, mean ± SD^g^	3.3 ± 0.8	3.1 ± 0.9	3.9 ± 1.4	5 ± 0
I	0	0	1	0
II	2	3	2	0
III	5	2	0	0
IV	4	4	3	0
V	1	0	6	9

Abbreviations: *NCI* no cognitive impairment, *AD* Alzheimer’s disease, *RADC* Rush Alzheimer’s Disease Center

^a^Age was different between *APOE3/3* NCI and *APOE3/3* AD groups, *APOE3/3* NCI and *APOE3/4* AD groups, and between *APOE3/4* NCI and *APOE3/4* AD groups; one-way ANOVA tests

^b^Proportion of males and females did not differ between groups; chi-squared test

^c^Education was different between *APOE3/3* NCI and *APOE3/3* AD groups; one-way ANOVA tests

^d^Average of *Z*-scores from 19 cognitive tests; scores provided by RADC

^e^Measure of neuritic plaque burden averaged across 5 cortex and hippocampus brain regions; scores provided by RADC

^f^Percent area of cortex occupied by amyloid beta; scores provided by RADC

^g^Braak stage was different between *APOE3/3* NCI and *APOE3/4* AD groups and between *APOE3/4* NCI and *APOE3/4* AD groups; one-way ANOVA tests

**Fig. 2:**
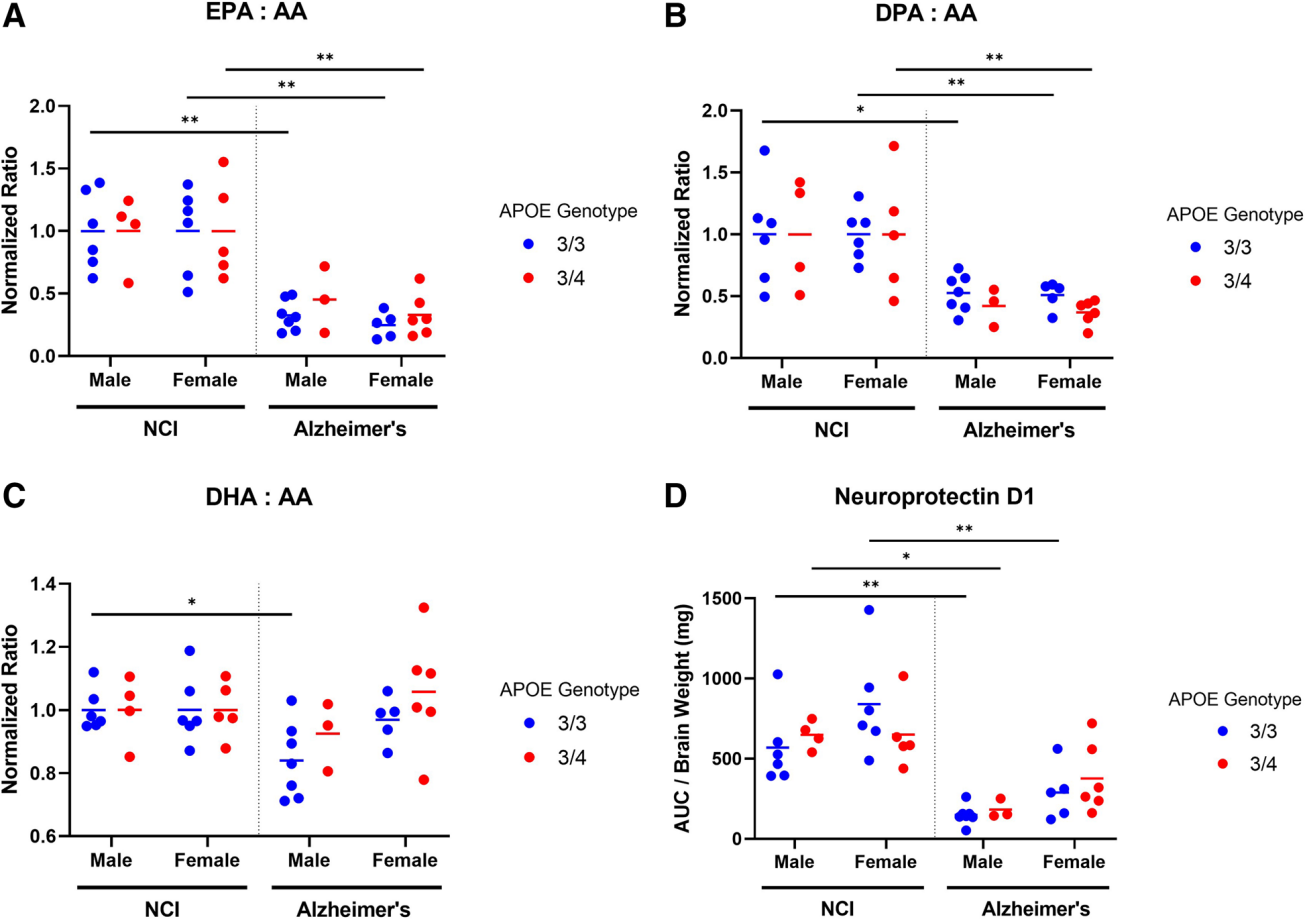
Dysregulation of bioactive lipids in the Alzheimer’s disease dementia brain evidenced by lower levels of ω-3: ω-6 fatty acids and pro-resolving lipid mediator NPD1. * *p* < 0.05, ** *p* < 0.01, *** *p* < 0.001; two-tailed Student’s *t* test. Abbreviations: AA, arachidonic acid; EPA, eicosapentaenoic acid; DHA, docosahexaenoic acid; DPA, docosapentaenoic acid; NPD1, neuroprotectin D1. Samples per group: NCI male 3/3 (*N*=6), NCI male 3/4 (*N*=4), NCI female 3/3 (*N*=6), NCI female 3/4 (*N*=5), AD male 3/3 (*N*=7), AD male 3/4 (*N*=3), AD female 3/3 (*N*=5), AD female 3/4 (*N*=6)

Lipidomic analysis of brains with AD dementia revealed a statistically significant reduction in the ratio of ω-3: ω-6 polyunsaturated fatty acids (PUFA) when compared to their corresponding NCI control groups (Fig. 2). This was most clearly evidenced by the EPA: AA and DPA: AA ratios, which were approximately 2–4-fold lower in brains with AD (Fig. 2A, B: * *p* < 0.05, ** *p* < 0.01, *** *p* < 0.001; two-tailed Student’s *t* test.). These associations occurred independently of sex or *APOE* genotype. The relative levels of PUFAs are depicted in Supplement Figure 1. In comparing DHA: AA ratios between NCI and AD groups in this cohort, significant reductions were only detected in males with AD who were *APOE* 3/3 (*p*-value < 0.05), whereas no differences were detected between the female NCI and AD groups (Fig. 2C). Neuroprotectin D1 (NPD1), a pro-resolving DHA metabolite, was also significantly lower across all AD subgroups (approximately 2–4-fold lower relative levels) except for in females carrying *APOE4*, who had marginally lower NPD1 levels compared to their respective NCI controls (Fig. 2D). These findings of lower ω-3: ω-6 ratios and NPD1 levels are consistent with previous clinical AD studies [17–20]. Furthermore, these findings underscore the importance of identifying the key pathways underlying lipid dysregulation in the AD dementia brain.

### APOE4 allele and failure to resolve neuroinflammation in AD dementia brain

**Fig. 3:**
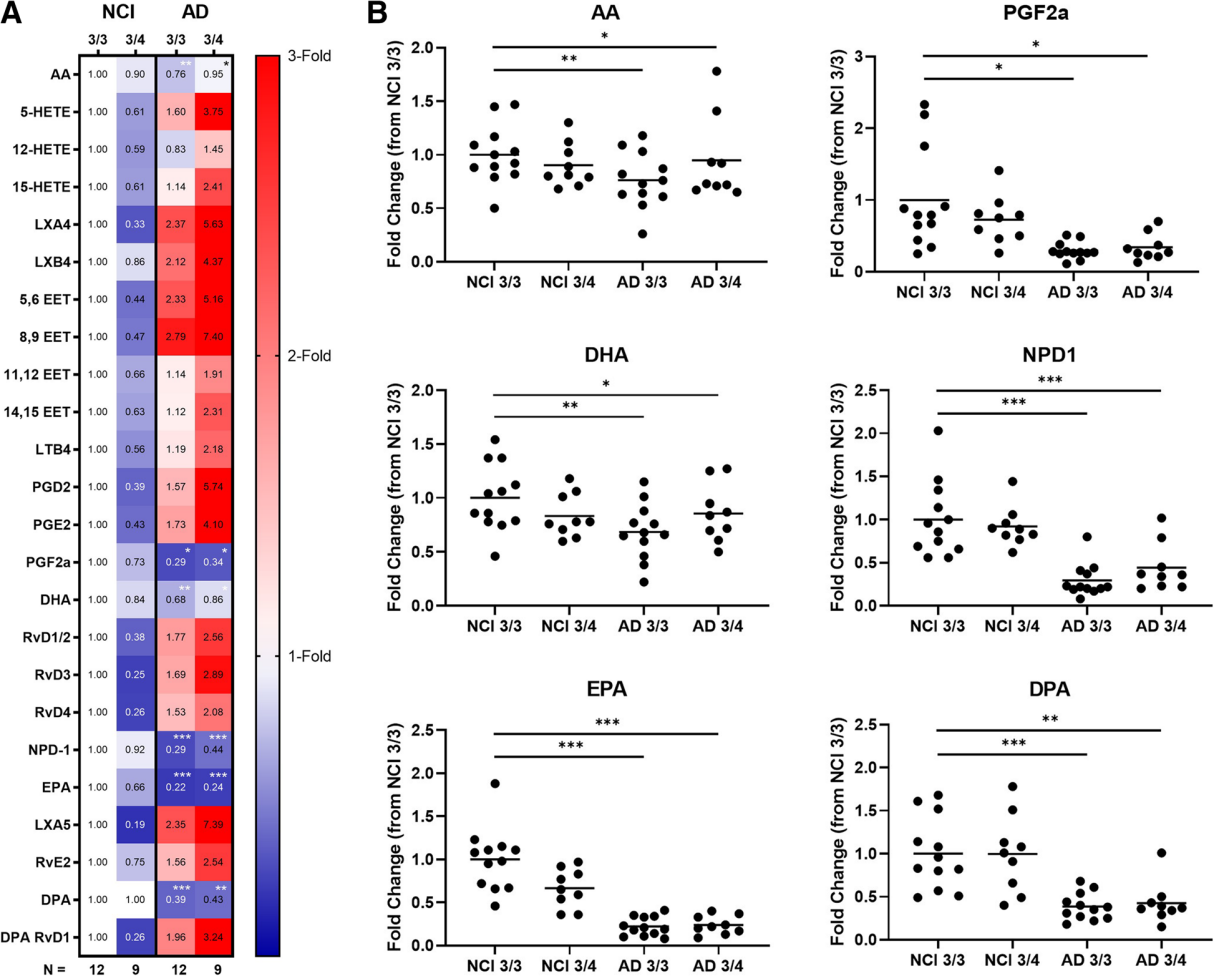
AD dementia brains from *APOE4* carriers display higher levels of pro-inflammatory and pro-resolving mediator lipids indicating chronic unresolved inflammation. * *p* < 0.05, ** *p* < 0.01, *** *p* < 0.001; p-values for β-estimates were derived from the regression model adjusted for age and education. Abbreviations: AA, arachidonic acid; 5-HETE, 5-hydroxyeicosatetraenoic acid; PGs, prostaglandins; TXB2, thromboxane B2; 12-HHT, 12-hydroxyheptadecatrienoic acid; LXs, lipoxins; LTB4, leukotriene B4; EPA, eicosapentaenoic acid; 5-HEPE, 5-hydroxyeicosapentaenoic acid; RvE3, resolvin E3; DHA, docosahexaenoic acid; 17-HDHA, 17-hydroxydocosahexaenoic acid; RvDs, D-series resolvins; NPD-1, neuroprotectin D1; DPA, docosapentaenoic acid

We have previously shown that *APOE4* with AD is associated with lower DHA: AA and EPA: AA ratios in the plasma and CSF after DHA supplementation compared to non-carriers [21]. Subsequently, we identified higher cPLA2 enzymatic activity in brains with *APOE4*, which was associated with higher levels of AA and its inflammatory metabolites [14]. Here, we investigated the association between these lipids and *APOE4* status in brain tissues. Lipid levels in cortical tissue from persons with AD and NCI were stratified by *APOE* genotype (*APOE* 3/3 or *APOE* 3/4). To understand the effect of *APOE* genotype on cortical tissue lipid composition, NCI *APOE* 3/3 was used as the reference control group, which subgroups were compared to. The data is summarized in Fig. 3, where values are represented as the mean fold-change relative to PUFA and eicosanoid levels found in NCI *APOE* 3/3. Blue cells signify mean fold-changes <1.00 and lower expression, while red cells denote mean fold-changes >1.00 and higher expression. Consistent with our initial findings, ω-3 fatty acids EPA, DHA and DPA, and NPD1 were downregulated in the AD dementia group after adjusting for age and education (Supplement Table 1). Contrary to NPD-1, all other detected SPMs metabolized from ω-3 fatty acids were higher in brains with AD dementia. These include RvD1/2, RvD3, RvD4, LXA5, RvE2, and DPA-derived RvD1. In brains with AD dementia, the presence of *APOE4* associated with greater elevations of these analytes compared to non-carriers.

In probing the bioactive lipid metabolites of ω-6 fatty acid AA, the majority of detected eicosanoids exhibited higher levels in brains with AD dementia. Pro-inflammatory bioactive lipids like prostaglandin D2 (PGD2), prostaglandin E2 (PGE2), leukotriene B4 (LTB4), and 5-HETE were all elevated. In this cohort, pro-resolving SPMs such as lipoxin A4 (LXA4), LXB4, and the epoxyeicosatrienoic acids (EETs) were also higher. Greater levels of these lipid mediators were more pronounced in those with AD dementia carrying the *APOE4* allele compared to non-carriers with AD. Prostaglandin-F2a (PGF2a) was lower across AD groups regardless of *APOE* genotype.

### Lipid mediators of inflammation are associated with clinical and neuropathology phenotypes

**Fig. 4:**
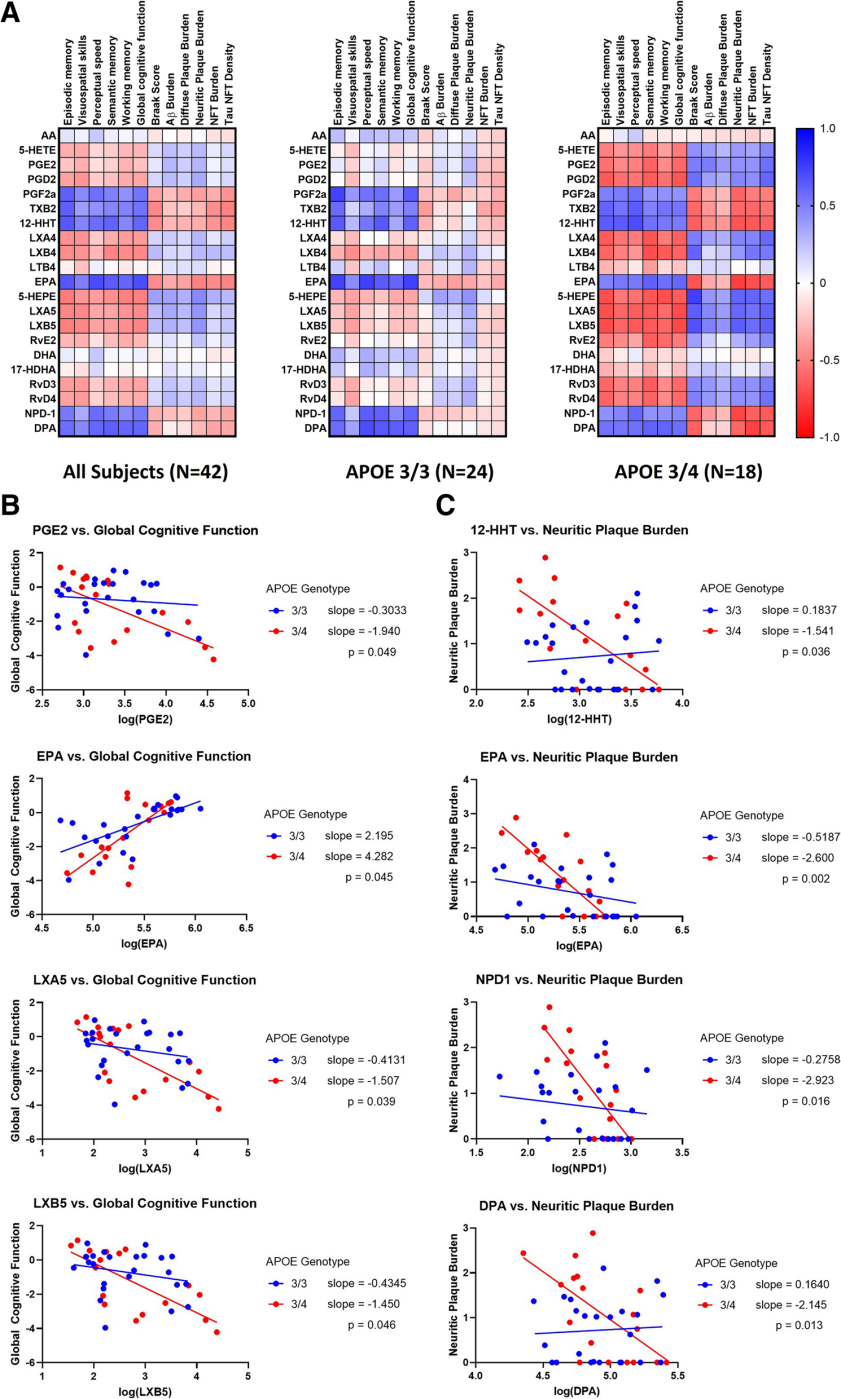
**A** Correlation matrices illustrate the relationships between cortical lipid levels and clinical and AD neuropathology phenotypes, which are exaggerated in *APOE4* carriers. Color-coded scale from -1.0 to 1.0 denotes the relative intensities of spearman correlation coefficients (*r*_*s*_). **B** Interaction effects between some of lipid mediator levels and *APOE4* on global cognitive function (**C**) and neuritic plaque burden. Abbreviations: AA, arachidonic acid; 5-HETE, 5-hydroxyeicosatetraenoic acid; PGs, prostaglandins; TXB2, thromboxane B2; 12-HHT, 12-hydroxyheptadecatrienoic acid; LXs, lipoxins; LTB4, leukotriene B4; EPA, eicosapentaenoic acid; 5-HEPE, 5-hydroxyeicosapentaenoic acid; RvE3, resolvin E3; DHA, docosahexaenoic acid; 17-HDHA, 17-hydroxydocosahexaenoic acid; RvDs, D-series resolvins; NPD-1, neuroprotectin D1; DPA, docosapentaenoic acid; NFT, Neurofibrillary tangles. *P* values represent the interaction of *APOE* genotype and lipid mediators on global cognitive function and neuritic plaque burden

To determine the impact of lipidome changes on clinical phenotypes, we evaluated the associations between brain lipidome markers and cognitive measures (global cognitive function, in addition to cognitive domains of episodic memory, working memory, semantic memory, perceptual speed, and visuospatial skills), as well as neuropathology markers. Using clinical scores, correlation matrices were produced to delineate relationships between lipid mediator levels and these phenotypic measurements. The results are summarized in Fig. 4, where blue cells signify positive correlations with Spearman correlation coefficients (*r*_*S*_) closer to 1, while red cells signify negative correlations with *r*_*S*_ values closer to −1. Correlational analyses across the entire study cohort (*N*=42) revealed strong positive correlations between ω-3 PUFAs and all cognitive function scores. In particular, higher EPA, DPA, and NPD-1 brain levels correlated with higher levels of cognitive functions. In contrast, EPA, DPA, and NPD-1 were negatively correlated with Braak staging and histological scores for Aβ and NFT burden. To a lesser degree, DHA and its hydroxylated derivative, 17-HDHA, also expressed positive correlations with cognitive functions and inverse relationships with histological disease burden. Other SPM metabolites of ω-3 PUFAs, including RvE2, RvD3, and RvD4 displayed weak inverse relationships with cognitive function and positive correlations with histologic disease markers.

The ω-6 PUFA AA showed a weak positive correlation with cognitive function scores and weak inverse relationships with Braak Stage and NFT pathologies. AA lipid metabolites with pro-inflammatory roles had varied trends. While PGD2, PGE2, 5-HETE, and LTB4 were negatively correlated with cognitive scores and positively correlated with histological disease state, an opposite relationship was observed for PGF2a and TXB2.

Eicosanoid lipids in brain tissues were significantly associated with measures of cognition and AD neuropathology after accounting for the major AD risk factors. Linear regression analyses revealed that associations between many lipids and global cognitive function persisted after adjusting for age, sex, and *APOE* genotype (Supplement Table 2). In a multivariate regression model adjusted for age, sex, and *APOE* genotype, EPA, PGD2, and 12-HHT were found to be strong predictors of global cognitive function (adjusted *R*^2^ = 0.644) (Supplement Table 3). EPA levels, when adjusted for age, sex, and *APOE* genotype, also exhibited significant associations with neuritic plaque burden (adjusted *R*^2^ = 0.407) and global AD pathology (adjusted *R*^2^ = 0.353). Furthermore, AA, 5-HEPE, and DPA were identified as strong predictors of overall Aβ burden in a multivariate regression model adjusted for age, sex, and *APOE* genotype (adjusted *R*^2^ = 0.330). Similar multivariate regression analyses between lipid mediators and tau-related pathologies identified AA and 5-HEPE as significant predictors of NFT burden (adjusted *R*^2^ = 0.381), and TXB2 as a predictor of tau NFT density (adjusted *R*^2^ = 0.255).

Comparing *APOE* 3/3 and *APOE* 3/4 brains revealed a clear influence of the *APOE4* allele (Fig. 4A). The correlations of lipid mediators with measures of cognition and AD pathology were stronger in *APOE4* carriers compared to *APOE3*, underscoring the influence of *APOE* genotype on the extent of neuroinflammation and disease severity. To test whether the associations between lipid levels and *APOE4* alleles had a significant interaction on cognitive function and neuropathology markers, interaction terms ((lipid levels) *(*APOE* genotype)) were incorporated into linear regression models with cognition or neuropathology scores as outcomes (Supplement Tables 4 and 5). The interaction effect between EPA, PGE2, LXB5, LXB4, and *APOE4* on global cognition was significant (*p* < 0.05), while PGD2, LTB4, and LXA4, 5-HEPE and RvD3 showed marginal significance (0.05 < *p* < 0.1) (Fig. 4B). The interaction effect between PGE2, PGD2, LXA5 and LXB5, and *APOE4* on Braak scores was significant, while marginal significance was observed for 5-HETE, 5-HEPE, LXA4, RvD3, RvD4, and NPD1. Finally, there was a significant interaction between NPD1, EPA, DPA and 12-HHT, and *APOE4* on the neuritic plaque burden (Fig. 4C).

### Altered enzymatic activity in lipid biosynthesis pathways in AD dementia

**Fig. 5:**
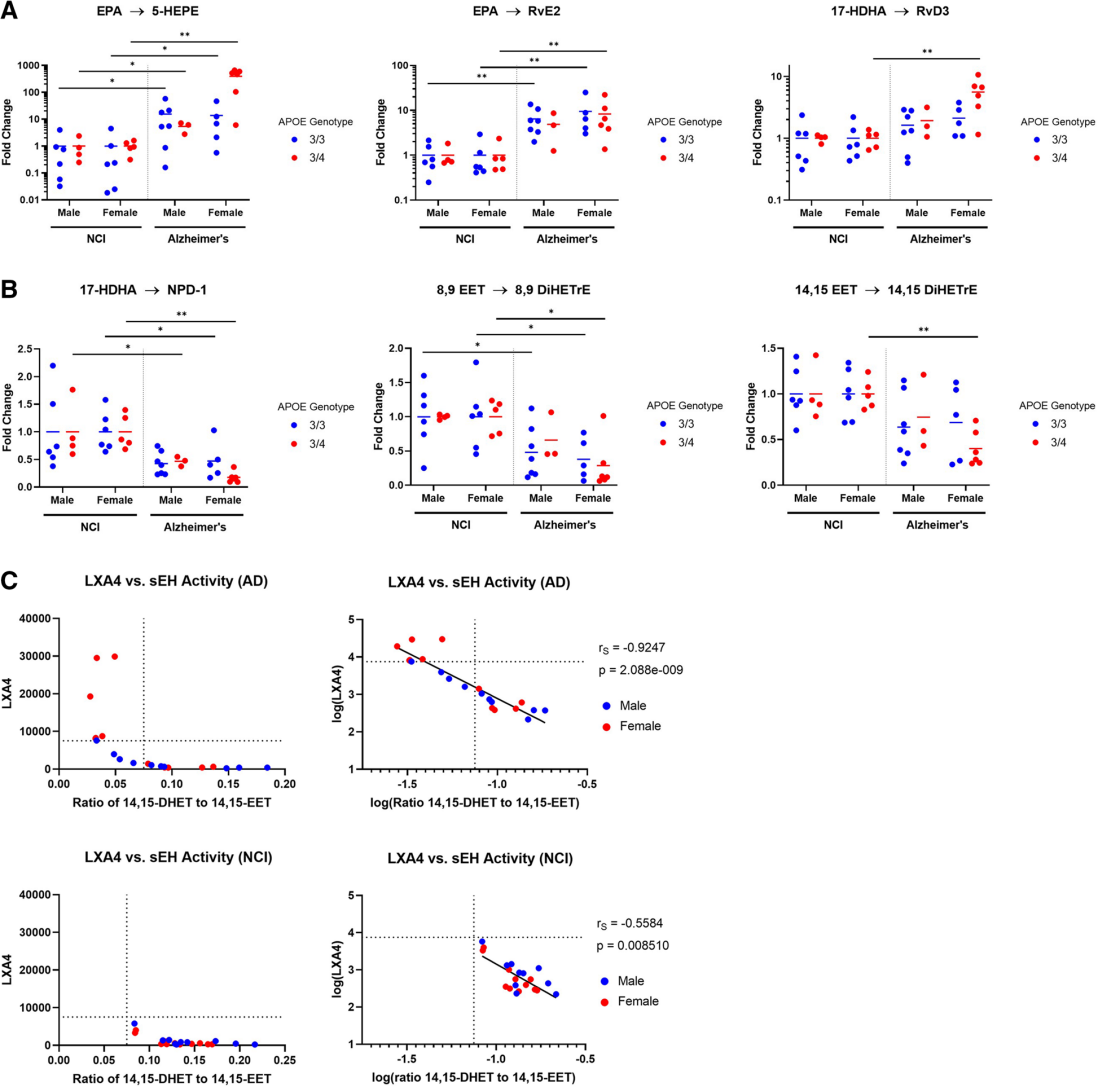
**A** Increased 5-LOX activity surrogates in brains with AD dementia. **B** Decreased soluble epoxide hydrolase (sEH) activity surrogates in brains with AD dementia. * *p* < 0.05, ** *p* < 0.01, *** *p* < 0.001; two-tailed Student’s *t* test. **C** Inverse relationship between sEH activity and LXA4 production in brains with AD and NCI on linear (left) and log-scale (right). EPA, eicosapentaenoic acid; HETEs, hydroxyeicosatetraenoic acids; HEPEs, hydroxyeicosapentaenoic acids; 17-HDHAs, 17-hydroxydocosahexaenoic acid, LXA4, lipoxin A4; EETs, epoxyeicosatrienoic acids; DHETs, dihydroxyeicosatrienoic acids; RvEs, E-series resolvins, RvDs, D-series resolvins; NPDs, neuroprotectins

To identify enzymes potentially responsible for the observed changes in lipid metabolism which could influence AD progression, comparative analyses of metabolite ratios were performed. Metabolite ratios served as indirect estimates of enzymatic activity and were calculated as the normalized AUC of a product lipid divided by that of its precursor (product: precursor). The lower ratio of EPA, DPA, and DHA to AA in persons with AD dementia compared with NCI suggest activation of cPLA2 [22]. There is also support for higher 5-LOX activity compared to age-matched NCI controls. This was evidenced by significantly higher metabolite ratios for conversions mediated by 5-LOX, including AA and EPA to their pro-inflammatory 5-hydroxy metabolites, 5-HETE and 5-HEPE (Fig. 5A). In the established lipid metabolism pathway, 5-HETE and 5-HEPE are further metabolized by 5-LOX to generate leukotrienes, which are also pro-inflammatory. Considering that 5-LOX activation is also required for lipoxin and resolvin biosynthesis, our results implicate 5-LOX activation as a likely driver for the increase of both pro-inflammatory and pro-resolving lipids observed in brains with AD. Comparing the conversion of 17-HDHA to RvD3, which is also mediated by 5-LOX, similar trends were observed where female *APOE4* carriers with AD displayed the greatest RvD3: 17-HDHA metabolite ratios compared to their NCI control group (>5-fold increase).

The enzymatic activity of soluble epoxide hydrolase (sEH) was also indirectly estimated using metabolite ratios. This enzyme mediates the formation of NPD-1 from 17-HDHA and catalyzes the breakdown of anti-inflammatory epoxyeicosatrienoic acids (EETs) to their less potent metabolites (DiHETrEs). Compared to persons with NCI, sEH-mediated metabolite ratios were significantly lower in brains with AD (Fig. 5B). Using the 14,15-DiHETrE: 14,15-EET ratio as a surrogate measure of relative sEH activity, an inverse relationship between estimated sEH activity and LXA4 levels was found in brains with AD (Fig. 5C). When comparing 14,15 DiHETrE: 14,15 EET ratios from NCI versus AD, a threshold of 0.075 was observed in persons with NCI. In contrast, approximately half of brain tissues from persons with AD exhibited ratios below this threshold. In brains with AD below this threshold, lower estimated sEH activity was associated with a sharp increase in LXA4 levels. Brains with AD having 14,15 DiHETrE: 14,15 EET ratios below 0.075 were also predominantly female, suggesting that hormonal effects may influence lipid metabolism. *APOE* genotype did not significantly influence sEH-mediated metabolite ratios (data not shown).

## Discussion

Although neuroinflammation has been implicated in AD clinical syndromes and is associated with greater disease severity, less is known regarding the role of brain PUFA metabolism and their immunomodulatory metabolites or the mechanisms by which they might accelerate disease progression by disrupting inflammatory homeostasis. Here, we show clear evidence of chronic neuroinflammation in brain tissue from persons with AD dementia, which is increased in those carrying one copy of the *APOE4* allele.

There are two major findings in this study. First, we identified a state of chronic unresolved inflammation in the dementia brain as revealed by elevated levels of both pro-inflammatory and pro-resolving mediators within a microenvironment, which are deleterious in the context of AD dementia because of their roles in neurodegeneration and tissue damage [23]. Specifically, we found the ratios of EPA:AA and DPA:AA were significantly lower in AD dementia brain tissues as compared to NCI controls. This result is consistent with prior lipidomic studies which have identified lower ω-3: ω-6 ratios in plasma as a risk factor for dementia [17–20]. Since ω-3 fatty acids like EPA, DHA, and DPA are precursors to a diversity of SPMs, lower ratios of ω-3: ω-6 PUFAs indicate chronic inflammation in the AD dementia brain. In addition, we found lower levels of pro-resolving DHA-derived NPD1 in brains with AD dementia, providing further evidence that dysregulation of free fatty acid content promotes chronic inflammation in AD by altering the generation of downstream lipid mediators. Whether this change in the brain lipidome is a cause or result of AD dementia cannot be inferred from this study, but in animal models, ω-3 deficiency has been linked to higher oxidative stress and lipid peroxidation, and lower synaptic plasticity, all of which may contribute to neurodegeneration and cognitive decline [5, 6, 24]. Similarly, in humans, lower blood levels of DHA and EPA predict a greater risk of AD dementia [25]. In cellular models, NPD1 induces neuronal survival and downregulates amyloidogenic processes such as Aβ42 peptide shedding and inflammatory gene expression in response to Aβ aggregation [6, 15, 26]. These effects are due in part to NPD1 activation of PPARγ, which counteracts inflammatory stimuli such as Aβ. In measuring the bioactive metabolites of ω-3 and ω-6 PUFAs, we observed elevated levels of pro-inflammatory lipids including PGD2, PGE2, LTB4, and 5-HETE in brains with AD. Contrary to what others have reported, we also found higher levels of SPMs like lipoxins and EETs, although lower levels of EETs have only been demonstrated in AD mouse models [27]. The differences in lipoxin levels, however, could likely be attributed to tissue-specific expression of SPMs since lower lipoxin levels in human brain tissue have only been demonstrated in the hippocampus [16], whereas these findings were not confirmed in human entorhinal cortex tissue [28]. In addition to SPM metabolites of AA, resolvins derived from EPA, DHA, and DPA were also increased despite the reduction of ω-3 PUFAs in brains with AD. We speculate that this increase in SPMs represents a compensatory mechanism to counterbalance increased AA metabolites such as PGD2, PGE2, LTB4, and 5-HETE seen in AD. One metabolic switch is the metabolism of 17-HDHA. In this context, brains with AD had higher RvD3 levels and lower NPD-1 levels, suggesting a likely increase in 5-LOX activity. This reasoning corresponds with increased LXA4 biosynthesis and could explain the observed inverse relationship between estimated sEH activity from metabolite ratios and LXA4 production. Elevated levels of inflammatory lipids must be counterbalanced by pro-resolving mediators within the brain microenvironment. The increase of SPMs such as LXA4 and RvD3 may be a result of lower glial cell sensitivity to these lipid mediators, which may be associated, in turn, with lower expression of their respective receptors, ALXR and GPR32. An effect of this sort, for example, would be similar to what is observed in individuals with diabetes who have higher circulating levels of insulin due to lower insulin sensitivity. In the context of this study, the preferential conversion of 17-HDHA towards RvD3 in AD as opposed to NPD1 could be a result of lower receptor expression or sensitivity, since RvD3 and NPD1 activate cells through different GPCRs.

The second major finding from this work was that brain tissue from *APOE4* persons with dementia was associated with increased levels of both inflammatory and pro-resolving lipid mediators and correlated with lower measures of cognitive function and more extensive AD pathology markers. Notably, *APOE4* showed significant interactions with lipid mediators on the Braak stage and neuritic plaques, more so than measures of brain amyloidosis. Although *APOE4* is known to have strong effects on brain amyloidosis, its interaction with lipid mediators explained performance on cognitive measures and the burden of neuritic plaques, implicating chronic unresolved inflammation as a mechanism that associates *APOE4* with cognitive decline. In addition, the increased catabolism of ω-3s can help explain why *APOE4* carriers with dementia are refractory to ω-3 supplementation [29, 30] and potentially, suggest that if there is a role for ω-3 supplements, it is in the prevention of this disease rather than treatment. Although *APOE4* is associated with a greater response to neuroinflammation and risk of developing AD, the mechanistic cause of this association is not fully elucidated. Among these pathways, we recently observed a failure to resolve cPLA2 activation by ApoE4 in cellular models, animal models, and human brains, driven by MAPK P38 signaling and involved both astrocytes and synaptosomes [14]. The findings here confirm an eicosanoid fingerprint of cPLA2 activation, or more likely, a failure to resolve its activation. It is possible that *APOE4* has similar effects on 5-LOX and sEH, however, the effect of *APOE4* on these pathways has not been delineated. Nonetheless, *APOE4* is known to have pleiotropic effects on AD onset and progression, including through altered Aβ metabolism and clearance, roles in tau pathology, and mitochondrial dysfunction [31, 32]. *APOE4* also promotes increased neuronal activation that is manifested by increased Ca^2+^ influx into neurons compared to *APOE2* or *APOE3* [33]. This could be another mechanism by which ApoE4 activates calcium-dependent lipid metabolizing enzymes such as cPLA2 and 5-LOX. Interestingly, we observed that persons with NCI carrying the *APOE4* allele exhibited lower levels of lipid mediators in the brain compared to non-*APOE4* carriers. This confirms the pathobiologic model that *APOE4* associates with a failure to resolve the inflammation as opposed to stimulating inflammation per se, and more importantly, that the effects of *APOE4* are modifiable. In this multiple-hit hypothesis of AD, *APOE4* carriers with chronic unresolved tissue inflammation, for example from eicosanoid lipidome activation, have a greater risk of developing AD dementia [34, 35]. The chronic inflammatory insult which predisposes *APOE4* carriers to AD dementia could result from a variety of factors. One potential driver is the expression of RNASE6, which encodes an antimicrobial peptide and has been linked to AD-related inflammatory genes, since it was recently shown to associate with worse memory in *APOE4* carriers and express at higher levels in the DLPFC of persons with AD [36–38]. Other examples include systemic inflammation, traumatic brain injury, or poor lifestyle in the context of chronic lower ω-3 consumption [39, 40]. Targeting brain inflammation by either prevention (such as avoidance of traumatic brain injury, addressing vascular risk factors, greater ω-3 dietary consumption), or by targeting the enzymes that activate lipid inflammation pathways (such as cPLA2) are promising strategies to reverse the progression of disease in *APOE4* carriers with or at risk of dementia.

A particular strength of this study is that we were able to correlate inflammatory bioactive lipid mediators with qualitative measures of cognitive function and post-mortem AD pathologic markers. Most notably, we found that anti-inflammatory ω-3 PUFAs EPA, DPA, and DHA-derived NPD-1 were positive correlated with cognitive scores (global cognitive function, as well as cognitive domains of episodic memory, working memory, semantic memory, perceptual speed, and visuospatial skills) and negatively associated with histologic markers of AD (Braak stage, Aβ, and NFT burden). Conversely, pro-inflammatory ω-6 metabolites PGD2, PGE2, 5-HETE, and LTB4 were negatively associated with cognition and positively associated with AD pathologies. There are several implications of these findings. Importantly, these correlations were most prominent in *APOE4* carriers, providing further evidence that the *APOE4* allele may increase the susceptibility to AD disease progression as a consequence of greater neuroinflammation.

In addition to describing the dysregulation of lipid metabolism in AD and the relationships between lipid markers and AD phenotypes, our analysis also identified potential novel therapeutic targets. In our analysis, we found that brains with AD exhibited higher surrogate measures of both cPLA2 and 5-LOX activities compared to age-matched NCI controls. This is supported by the significant increase in metabolite ratios mediated by these enzymes, such as the conversions of AA and EPA to their pro-inflammatory 5-hydroxy metabolites, 5-HETE and 5-HEPE. Given that 5-LOX activation is also required for lipoxin and resolvin biosynthesis, our results implicate cPLA2 and 5-LOX activities as important drivers for the increase of both pro-inflammatory and pro-resolving lipids observed in brains with AD. These observations still need to be validated in clinical trials. 

## Limitations

There are a few limitations of this study. The lipidomic data were collected from a single brain region, the dorsolateral prefrontal cortex (DLPFC), and measures may differ from other brain regions in terms of lipid metabolism. Additionally, blood samples were not obtained from the same cohort for comparison of lipid profiles between blood and brain. We also acknowledge that in some of our statistical analyses, the sample sizes may be too small to detect small differences. Furthermore, it is likely that greater differences would be achieved by using brain tissues collected from *APOE4* homozygotes, since this genotype is known to have stronger disease phenotypes than *APOE4* heterozygotes [41]. Furthermore, some bioactive lipids known to be derived from PUFAs went undetected (e.g., maresins), and it is likely that the lipid signaling pathway is not fully elucidated as there are probably other enzymes and metabolites involved that have not yet been identified. Future studies should include quantification of phospholipid species, and the expression levels for lipid mediator receptors to paint a more complete picture of the lipidome across different regions of the human brain. For similar reasons, non-esterified ω-3 precursors such as alpha-linolenic acid (ALA) and docosatetraenoic acid (DTA) should be incorporated in future lipidomic studies, although the endogenous production of ω-3 PUFAs such as DHA is known to be limited [42]. Given the hypothesis that the individual eicosanoid lipid mediators are associated with clinical and neuropathologic markers of AD, the lipids were run in individual linear models without adjusting for multiple comparisons. Finally, the *APOE4* group with dementia appeared to have a more severe disease (by Braak stage, neuritic plaques, or global cognitive function indices) and it is possible that disease severity and not *APOE4* per se explains some of the associations we observed. The sample size of this cohort is small and prohibits adjusting for these variables. More importantly, it is plausible that *APOE4*’s effect in those with dementia is mediated by worsening the severity of the disease.

## Materials and methods

### Clinical data, pathologic data, and brain tissues

Clinical and pathologic data, along with frozen postmortem brain tissues from the DLPFC, were obtained from deceased participants who were enrolled in the Religious Order Study (ROS). The study was approved by the Institutional Review Board of Rush University Medical Center and the University of Southern California. Briefly, eligible participants had no known dementia at the time of enrollment and agreed to annual clinical assessments as well as to brain donation at the time of death. Clinical assessments were performed annually, from which cognitive and other data were generated. Neuropsychological data were summarized into composite measures of global cognition and five cognitive domains, including episodic memory, working memory, semantic memory, perceptual speed, and visuospatial skills. Cognitive data proximate to death were used in analyses. Dementia classification was conducted by a clinician with expertise in aging, after a review of all cognitive and other clinical data, but blinded to pathologic data. *APOE4* genotyping was done using standard methods. At the time of death, a brain autopsy was performed. The average postmortem interval of the study is about 8 hours. A detailed neuropathologic examination was conducted blinded to all clinical data, including for AD pathology. Measures based on a modified Bielschowsky stain of neuritic plaques, diffuse plaques, and neurofibrillary tangles (NFTs) were used to form composite measures of global AD pathology. In addition, immunohistochemical measures yielded data on the Aβ burden and tau NFT density.

### Lipid extraction and LC-MS quantification

The extraction and detection of lipids from ROS brain tissues by LCMS/MS were performed at the University of Southern California. Lipids were extracted from brain tissues and analyzed using liquid chromatography-mass spectrometry (LC-MS) and quantified using multiple reaction monitoring (MRM) mass to transition ion signatures. Specifically, lipids were extracted from homogenized DLPFC tissue (approximately 50–100 mg) by adding 500 μL methanol. To this suspension, 10 μL of internal standards containing a mixture of d5-RvD2, d8-5S-HETE, d4-PGE2, d5-LXA4, and d4-LTB4 were added. After thorough homogenization using a TissueLyser (Qiagen) at 30 Hz for 5-min intervals, the samples were centrifuged at 10,000 rpm for 5 min at 4^o^C. The supernatant was removed and diluted with water to a final concentration of 10% methanol. Samples were further extracted using Strata X 33μm Polymeric Reverse Phase cartridge separation. Cartridges were pre-conditioned with methanol (1 mL) followed by water (1 mL) before the clarified samples were layered onto the cartridge. The lipid components were eluted using 500 μL methanol, which was then evaporated to dryness under a steady stream of filtered and dried N_2_ gas. The dried residues were then reconstituted with 50% methanol (50 μL) and centrifuged to remove any undissolved debris. The analytes were separated and quantified by Agilent 1290 UPLC linked onto a QTRAP Sciex API6500+ LCMS/MS system (Sciex). For separation, a Poroshell 120 EC-C18 column (2.7 μm, 4.6 ×100 mm, Agilent) was used with the following binary mobile phase: water + 0.01% FA (mobile phase A) and methanol + 0.01% FA (mobile phase B). The gradient [time (%A/%B)] was programmed as follows: 0 min (80/20)–0.1 min (50/50)–2 min (50/50)–11 min (20/80)–14.5 min (20/80)–14.6 min (2/98)–20 min (2/98)–20.1 min (80/20)–23 min (80/20). An injection volume of 5 μL was used, and the flow rate was held at 0.5 mL/min while the column temperature was maintained at 40^o^C. Each of the targeted analytes was identified and quantified using their unique MRM signatures (Supplement Table 6) in negative mode (ESI-), in conjunction with deuterated synthetic standards (Cayman).

### Analyses

Analytes of interest include AA-derived pro-inflammatory lipid metabolites such as leukotrienes (LTs), prostaglandins (PGs), and thromboxanes (TBs), in addition to specialized pro-resolving mediators (SPMs) like lipoxins (LXs), resolvins (RsVs), neuroprotectins (NPDs) and maresins (MrSs). Peak selections were revised manually, and the resulting AUC values were normalized by the weight of tissue used for lipid extraction. Additionally, the normalized AUC values were adjusted by internal standard to account for batch-to-batch variability and sample loss during processing.

Lipid levels were compared between persons with confirmed Alzheimer’s disease (AD) and age-matched controls without clinical signs of neurological deficits, which were classified as non-cognitive impaired (NCI). In addition. samples were sub-classified according to *APOE* genotype and sex. To assess enzymatic activity, metabolite ratios were calculated as the normalized AUC of the product lipid divided by that of its precursor. Fold change values were calculated by setting the mean lipid levels for each NCI control group equal to 1.0 and comparing each brain with AD to its respective control group having the same sex and *APOE* genotype.

#### Data analysis

The relative levels of these lipids were quantified as acquired peak area normalized by the weight (mg) of brain tissue examined, and persons with NCI were used as a reference control. Group comparisons used a two-tailed Student’s *t* test. Linear models were used to study associations of lipid mediators and clinical/neuropathological outcomes with sex, age, or *APOE* genotype as covariates. Correlation matrices were produced to delineate relationships between lipid mediator levels and clinical or neuropathology measurements using Spearman correlation coefficients. To test whether the associations between lipid levels and *APOE4* alleles had a significant interaction on cognitive function and neuropathology markers, interaction terms ((lipid levels) *(*APOE* genotype)) were incorporated into linear regression models with cognition or neuropathology scores as outcomes. *P* < 0.05 was defined as significant, while *p*<0.1 was defined as marginally significant. The programs R, SPSS, and GraphPad Prism were used to perform the statistical analysis and generate the figures.

## Supplementary Information

**Additional file 1: Supplement Figure 1.** Comparison of relative PUFA levels in brains with NCI and AD dementia stratified by sex. **Supplement Table 1.** Linear regression analyses of lipid mediator levels compared to NCI *APOE* 3/3. **Supplement Table 2.** Linear regression analyses of lipid mediator levels vs. global cognitive function. **Supplement Table 3.** Multivariate linear regression analyses of lipid mediator levels vs. clinical phenotype measurements. **Supplement Table 4.** Interaction p-value of *APOE4* and lipid mediators on cognitive functions. **Table 5.** Interaction p-value of *APOE4* and lipid mediators on AD neuropathology markers. **Supplement Table 6.** LC-MS Assay MRM Signatures.

## Data Availability

Lipidomics data have been deposited on the ROS RADC database: https://www.radc.rush.edu.
