# Supplementary material for: Eicosanoid lipidome activation in post-mortem brain tissues of individuals with APOE4 and Alzheimer’s dementia
Source: Alzheimers Res Ther. 2022 Oct 11;14:152. doi: 10.1186/s13195-022-01084-7 (PMC9552454; doi:10.1186/s13195-022-01084-7)
Supplement: Supplementary file 1 — Additional file 1: Supplement Figure 1. Comparison of relative PUFA levels in brains with NCI and AD dementia stratified by sex. Supplement Table 1. Linear regression analyses of lipid mediator levels compared to NCI APOE 3/3. Supplement Table 2. Linear regression analyses of lipid mediator levels vs. global cognitive function. Supplement Table 3. Multivariate linear regression analyses of lipid mediator levels vs. clinical phenotype measurements. Supplement Table 4. Interaction p-value of APOE4 and lipid mediators on cognitive functions. Table 5. Interaction p-value of APOE4 and lipid mediators on AD neuropathology markers. Supplement Table 6. LC-MS Assay MRM Signatures. [file 13195_2022_1084_MOESM1_ESM.docx]

**Supplement Figure 1: Comparison of relative PUFA levels in brains with NCI and AD dementia stratified by sex.**

Abbreviations: AA, arachidonic acid; DHA, docosahexaenoic acid; EPA, eicosapentaenoic acid; RvE2, resolvin E2; DPA, docosapentaenoic acid; NCI, no cognitive impairment.

Significance levels determined from two-tailed Student’s t-test.

^*^ p-value < 0.05

^**^ p-value < 0.01

^***^ p-value < 0.001

| **Supplement Table 1: Linear regression analyses of lipid mediator levels compared to NCI APOE 3/3** | | | | | | |
| --- | --- | --- | --- | --- | --- | --- |
|  |  | **Standardized β Coefficients** | | | | |
| **Covariate** |  | **NCI**  **APOE 3/4** | **AD**  **APOE 3/3** | **AD**  **APOE 3/4** | **Age (years)** | **Education (years)** |
| AA |  | -0.073 | -0.380^**^ | -0.320^*^ | 0.026^**^ | -0.006 |
| 5-HETE |  | -0.429 | 0.953 | 0.042 | 0.151^*^ | -0.030 |
| 12-HETE |  | -0.316 | 0.110 | -0.310 | 0.022 | -0.025 |
| 15-HETE |  | -0.222 | 0.451 | -0.193 | 0.092 | -0.027 |
| LXA4 |  | -0.917 | 1.588 | 0.148 | 0.308^*^ | -0.015 |
| LXB4 |  | -0.643 | 0.852 | 0.229 | 0.157^*^ | 0.019 |
| 5,6 EET |  | -0.801 | 1.610 | -0.021 | 0.274^*^ | 0.020 |
| 8,9 EET |  | -0.920 | 2.214 | 0.712 | 0.383^*^ | 0.033 |
| 11,12 EET |  | -0.173 | 0.368 | -0.352 | 0.053 | -0.063 |
| 14,15 EET |  | -0.205 | 0.367 | -0.252 | 0.091 | -0.027 |
| LTB4 |  | -0.451 | 0.227 | -0.392 | 0.107^*^ | 0.013 |
| PGD2 |  | -0.657 | 1.365 | 0.599 | 0.236 | -0.080 |
| PGE2 |  | -0.509 | 1.160 | 0.190 | 0.197^*^ | -0.037 |
| PGF2a |  | -0.064 | -0.636^*^ | -0.644^*^ | 0.000 | 0.006 |
| DHA |  | -0.044 | -0.417^**^ | -0.384^*^ | 0.023^**^ | -0.011 |
| RvD1/2 |  | -0.419 | 1.017 | 0.018 | 0.109 | -0.059 |
| RvD3 |  | -0.547 | 1.135 | -0.338 | 0.143 | -0.086 |
| RvD4 |  | -0.557 | 0.661 | -0.527 | 0.115 | -0.054 |
| NPD-1 |  | -0.123 | -0.770^***^ | -0.762^***^ | 0.016 | 0.001 |
| EPA |  | -0.024 | -0.815^***^ | -0.797^***^ | 0.006 | -0.008 |
| LXA5 |  | -1.019 | 1.686 | 1.383 | 0.4018^*^ | -0.174 |
| RvE2 |  | -0.533 | 0.440 | 0.292 | 0.073 | 0.045 |
| DPA |  | 0.190 | -0.717^***^ | -0.623^**^ | 0.009 | -0.014 |
| DPA RvD1 |  | -0.714 | 0.601 | 0.328 | 0.197^*^ | -0.030 |

Abbreviations: AA, arachidonic acid; HETEs hydroxyeicosatetraenoic acids; LXs, lipoxins; EETs, epoxyeicosatrienoic acids, LTB4, leukotriene B4; PGs, prostaglandins; DHA, docosahexaenoic acid; RvDs, D-series resolvins; NPD-1, neuroprotectin D1; EPA, eicosapentaenoic acid; RvE2, resolvin E2; DPA, docosapentaenoic acid.

Significance levels and β-coefficients reported for multivariate model with mean fold-change in lipid levels from NCI APOE 3/3 as the dependent variable, with age (years) and education (years) as covariates.

^*^ p-value < 0.05

^**^ p-value < 0.01

^***^ p-value < 0.001

| **Supplement Table 2: Linear regression analyses of lipid mediator levels vs. global cognitive function** | | | | | | |
| --- | --- | --- | --- | --- | --- | --- |
|  |  | **Standardized β Coefficients** | | | | |
| **Covariate** |  | **Model 1^a^** | **Model 2^b^** | **(Age)** | **(Sex)** | **(*APOE*)** |
| AA |  | 0.002 | 0.201 | -0.568^***^ | 0.120 | -0.051 |
| 5-HETE |  | -0.437^**^ | -0.268 | -0.417^**^ | 0.025 | -0.388 |
| PGE2 |  | -0.372^*^ | -0.221 | -0.448^**^ | 0.033 | -0.056 |
| PGD2 |  | -0.437^**^ | -0.285 | -0.420^**^ | 0.044 | -0.056 |
| PGF2a |  | 0.531^***^ | 0.413^**^ | -0.365^*^ | 0.131 | -0.055 |
| TXB2 |  | 0.495^**^ | 0.396^**^ | -0.334^*^ | 0.172 | -0.092 |
| 12-HHT |  | 0.562^***^ | 0.496^**^ | -0.296^*^ | 0.223 | -0.069 |
| LXA4 |  | -0.438^**^ | -0.269 | -0.405^**^ | 0.052 | -0.073 |
| LXB4 |  | -0.509^**^ | -0.353^*^ | -0.356^*^ | 0.037 | -0.098 |
| LTB4 |  | -0.276 | -0.108 | -0.481^**^ | 0.055 | -0.055 |
| EPA |  | 0.675^***^ | 0.573^***^ | -0.311^*^ | 0.148 | -0.021 |
| 5-HEPE |  | -0.490^**^ | -0.329^*^ | -0.355^*^ | 0.046 | -0.108 |
| LXA5 |  | -0.473^**^ | -0.296 | -0.383^*^ | 0.065 | -0.064 |
| LXB5 |  | -0.494^**^ | -0.325^*^ | -0.369^*^ | 0.071 | -0.067 |
| RvE3 |  | -0.429^**^ | -0.229 | -0.415^**^ | 0.047 | -0.049 |
| DHA |  | 0.062 | 0.232 | -0.562^***^ | 0.136 | -0.046 |
| 17-HDHA |  | -0.160 | -0.044 | -0.505^**^ | 0.076 | -0.050 |
| RvD3 |  | -0.388^*^ | -0.230 | -0.424^**^ | 0.054 | -0.088 |
| RvD4 |  | -0.357^*^ | -0.215 | -0.438^**^ | 0.050 | -0.091 |
| NPD-1 |  | 0.451^**^ | 0.460^**^ | -0.398^**^ | 0.232 | -0.107 |
| DPA |  | 0.567^***^ | 0.536^***^ | -0.385^**^ | 0.216 | -0.060 |

Abbreviations: AA, arachidonic acid; 5-HETE, 5-hydroxyeicosatetraenoic acid; PGs, prostaglandins; TXB2, thromboxane B2; 12-HHT, 12-hydroxyheptadecatrienoic acid; LXs, lipoxins; LTB4, leukotriene B4; EPA, eicosapentaenoic acid; 5-HEPE, 5-hydroxyeicosapentaenoic acid; RvE3, resolvin E3; DHA, docosahexaenoic acid; 17-HDHA, 17-hydroxydocosahexaenoic acid; RvDs, D-series resolvins; NPD-1, neuroprotectin D1; DPA, docosapentaenoic acid.

^a^ Model 1 is a univariate model with global cognitive function as the dependent variable and a single lipid analyte as the independent variable.

^b^ Model 2 is a multivariate model with global cognitive function as the dependent variable, with the covariates being a single lipid analyte, age, sex, and *APOE* genotype.

^*^ p-value < 0.05

^**^ p-value < 0.01

^***^ p-value < 0.001

| **Supplement Table 3: Multivariate linear regression analyses of lipid mediator levels vs. clinical phenotype measurements** | | | | | | |
| --- | --- | --- | --- | --- | --- | --- |
|  | **Standardized β Coefficients** | | | | | |
| **Covariate** | **Model 1^a^** | **Model 2^b^** | **Model 3^c^** | **Model 4^d^** | **Model 5^e^** | **Model 6^f^** |
| EPA | 0.433^**^ | -0.516^***^ | -0.453^**^ | NA | NA | NA |
| PGD2 | -0.301^**^ | NA | NA | NA | NA | NA |
| 12-HHT | 0.300^*^ | NA | NA | NA | NA | NA |
| AA | NA | NA | NA | -0.816^**^ | -0.561^***^ | NA |
| 5-HEPE | NA | NA | NA | 0.586^**^ | 0.389^*^ | NA |
| DPA | NA | NA | NA | 0.460^*^ | NA | NA |
| TXB2 | NA | NA | NA | NA | NA | -0.406^*^ |
| Age | -0.132 | 0.109 | 0.187 | 0.276 | 0.382^*^ | 0.265 |
| Sex | 0.168 | -0.249 | -0.265^*^ | -0.137 | 0.004 | -0.136 |
| APOE | -0.051 | 0.220 | 0.133 | 0.225 | 0.054 | 0.048 |

Abbreviations: EPA, eicosapentaenoic acid; PGD2, prostaglandin D2; 12-HHT, 12-hydroxyheptadecatrienoic acid; AA, arachidonic acid; 5-HEPE, 5-hydroxyeicosapentaenoic acid; DPA, docosapentaenoic acid; TXB2, thromboxane B2; NA, not applicable.

^a^ Model 1 is a multivariate model with global cognitive function as the dependent variable, with the covariates being EPA, PGD2, 12-HHT, age, sex, and *APOE* genotype.

^b^ Model 2 is a multivariate model with neuritic plaque burden as the dependent variable, with the covariates being EPA, age, sex, and *APOE* genotype.

^c^ Model 3 is a multivariate model with global AD pathology as the dependent variable, with the covariates being EPA, age, sex, and *APOE* genotype.

^d^ Model 4 is a multivariate model with Aβ burden as the dependent variable, with the covariates being AA, 5-HEPE, DPA, age, sex, and *APOE* genotype.

^e^ Model 5 is a multivariate model with NFT burden as the dependent variable, with the covariates being AA, 5-HEPE, age, sex, and *APOE* genotype.

^f^ Model 6 is a multivariate model with tau NFT density as the dependent variable, with the covariates being TXB2, age, sex, and *APOE* genotype.

^*^ p-value < 0.05

^**^ p-value < 0.01

^***^ p-value < 0.001

| **Supplement Table 4:** Interaction p-value of *APOE4* and lipid mediators on cognitive functions | | | | | | | |
| --- | --- | --- | --- | --- | --- | --- | --- |
|  | **Global Cognitive Function** | **Episodic Memory** | **Working Memory** | **Semantic Memory** | **Perceptual Speed** | **Visuospatial Skills** | **Braak Score** |
| **AA** | 0.256 | 0.415 | 0.225 | 0.236 | 0.523 | 0.932 | 0.469 |
| **5-HETE** | 0.159 | 0.168 | **0.096** | **0.093** | 0.403 | 0.860 | 0.145 |
| **PGE2** | **0.049** | **0.035** | **0.043** | **0.033** | 0.253 | 0.439 | **0.039** |
| **PGD2** | 0.062 | **0.056** | **0.032** | **0.054** | 0.275 | 0.565 | **0.041** |
| **PGF2a** | 0.556 | 0.588 | 0.293 | 0.477 | 0.772 | 0.983 | 0.368 |
| **TXB2** | 0.217 | 0.333 | **0.067** | 0.224 | 0.310 | 0.804 | 0.699 |
| **12-HHT** | 0.418 | 0.494 | 0.146 | 0.655 | 0.898 | 0.489 | 0.386 |
| **LXA4** | 0.097 | **0.093** | **0.043** | **0.057** | 0.428 | 0.626 | 0.128 |
| **LXB4** | 0.667 | 0.576 | 0.149 | 0.625 | 0.343 | 0.698 | 0.456 |
| **LTB4** | 0.126 | **0.095** | 0.106 | **0.059** | 0.322 | 0.720 | 0.157 |
| **EPA** | **0.045** | 0.235 | **0.031** | 0.488 | 0.436 | 0.365 | 0.168 |
| **5-HEPE** | 0.132 | 0.111 | **0.066** | 0.130 | 0.924 | 0.841 | 0.140 |
| **LXA5** | **0.039** | **0.042** | **0.024** | **0.053** | 0.352 | 0.460 | **0.040** |
| **LXB5** | **0.046** | **0.055** | **0.020** | **0.060** | 0.417 | 0.509 | **0.033** |
| **RvE3** | 0.161 | 0.144 | **0.053** | **0.055** | 0.679 | 0.783 | 0.224 |
| **DHA** | 0.453 | 0.386 | 0.482 | 0.222 | 0.618 | 0.994 | 0.427 |
| **17-HDHA** | **0.063** | **0.059** | **0.087** | **0.033** | 0.101 | 0.408 | 0.198 |
| **RvD3** | **0.074** | **0.065** | **0.043** | **0.052** | 0.288 | 0.580 | **0.077** |
| **RvD4** | **0.072** | **0.053** | **0.061** | **0.056** | 0.251 | 0.550 | 0.134 |
| **NPD-1** | 0.156 | 0.225 | 0.119 | 0.657 | 0.774 | 0.205 | **0.087** |
| **DPA** | 0.431 | 0.555 | 0.157 | 0.847 | 0.188 | 0.682 | 0.215 |

p ≤ 0.1 are in bold, p < 0.05 are highlighted. Abbreviations: AA, arachidonic acid; 5-HETE, 5-hydroxyeicosatetraenoic acid; PGs, prostaglandins; TXB2, thromboxane B2; 12-HHT, 12-hydroxyheptadecatrienoic acid; LXs, lipoxins; LTB4, leukotriene B4; EPA, eicosapentaenoic acid; 5-HEPE, 5-hydroxyeicosapentaenoic acid; RvE3, resolvin E3; DHA, docosahexaenoic acid; 17-HDHA, 17-hydroxydocosahexaenoic acid; RvDs, D-series resolvins; NPD-1, neuroprotectin D1; DPA, docosapentaenoic acid.

| **Table 5:** Interaction p-value of *APOE4* and lipid mediators on AD neuropathology markers | | | | | | |
| --- | --- | --- | --- | --- | --- | --- |
|  | **Global AD Pathology** | **Neuritic Plaque**  **Burden** | **Diffuse Plaque**  **Burden** | **NFT Burden** | **Aβ Burden** | **Tau NFT Density** |
| **AA** | 0.957 | 0.480 | 0.729 | 0.516 | 0.939 | 0.451 |
| **5-HETE** | 0.527 | 0.916 | 0.661 | 0.277 | 0.442 | 0.558 |
| **PGE2** | 0.354 | 0.831 | 0.429 | 0.186 | 0.259 | 0.908 |
| **PGD2** | 0.384 | 0.766 | 0.651 | 0.131 | 0.431 | 0.850 |
| **PGF2a** | 0.388 | 0.150 | 0.596 | 0.167 | 0.811 | 0.168 |
| **TXB2** | 0.324 | 0.184 | 0.993 | 0.251 | 0.582 | 0.340 |
| **12-HHT** | **0.088** | **0.036** | 0.559 | 0.154 | 0.365 | **0.054** |
| **LXA4** | 0.520 | 0.868 | 0.761 | 0.221 | 0.411 | 0.747 |
| **LXB4** | 0.496 | 0.860 | 0.275 | 0.795 | 0.588 | 0.251 |
| **LTB4** | 0.651 | 0.732 | 0.521 | 0.354 | 0.372 | 0.503 |
| **EPA** | **0.100** | **0.002** | 0.907 | 0.384 | 0.946 | 0.201 |
| **5-HEPE** | 0.649 | 0.700 | 0.854 | 0.588 | 0.906 | 0.915 |
| **LXA5** | 0.268 | 0.497 | 0.566 | 0.131 | 0.313 | 0.728 |
| **LXB5** | 0.209 | 0.401 | 0.589 | **0.076** | 0.250 | 0.658 |
| **RvE3** | 0.657 | 0.732 | 0.457 | 0.457 | 0.539 | 0.204 |
| **DHA** | 0.781 | 0.285 | 0.871 | 0.698 | 0.805 | 0.580 |
| **17-HDHA** | 0.361 | 0.760 | 0.395 | 0.257 | 0.266 | 0.834 |
| **RvD3** | 0.335 | 0.639 | 0.661 | 0.114 | 0.172 | 0.853 |
| **RvD4** | 0.598 | 0.876 | 0.834 | 0.303 | 0.354 | 0.963 |
| **NPD-1** | 0.121 | **0.016** | 0.985 | 0.124 | 0.772 | **0.025** |
| **DPA** | 0.184 | **0.013** | 0.927 | 0.349 | 0.648 | 0.108 |

p ≤ 0.1 are in bold, p < 0.05 are highlighted. Abbreviations: AA, arachidonic acid; 5-HETE, 5-hydroxyeicosatetraenoic acid; PGs, prostaglandins; TXB2, thromboxane B2; 12-HHT, 12-hydroxyheptadecatrienoic acid; LXs, lipoxins; LTB4, leukotriene B4; EPA, eicosapentaenoic acid; 5-HEPE, 5-hydroxyeicosapentaenoic acid; RvE3, resolvin E3; DHA, docosahexaenoic acid; 17-HDHA, 17-hydroxydocosahexaenoic acid; RvDs, D-series resolvins; NPD-1, neuroprotectin D1; DPA, docosapentaenoic acid.

**Supplement Table 6: LC-MS Assay MRM Signatures**

| **Compound** | **Q1 (*m/z*)** | **Q3 (*m/z*)** | **RT (min)** | **DP (V)** | **CE (V)** | **CXP (V)** | **Internal Standard** |
| --- | --- | --- | --- | --- | --- | --- | --- |
| d8-5S-HETE | 327.3 | 116.1 | 17.5 | -80 | -17 | -10 | N/A |
| d4-LTB4 | 339.3 | 197.2 | 14.2 | -80 | -22 | -13 | N/A |
| d4-PGE2 | 355.3 | 193.2 | 11.3 | -80 | -25 | -16 | N/A |
| d5-LXA4 | 356.3 | 115.2 | 11.8 | -80 | -19 | -14 | N/A |
| d5-RvD2 | 380.3 | 141.2 | 11.3 | -80 | -23 | -14 | N/A |
| AA | 303.3 | 259.1 | 18.7 | -100 | -16 | -18 | d8-5S-HETE |
| 5-HETE | 319.2 | 115.1 | 17.7 | -80 | -21 | -12 | d8-5S-HETE |
| 12-HETE | 319.2 | 179.1 | 17.6 | -80 | -21 | -12 | d8-5S-HETE |
| 15-HETE | 319.2 | 219.1 | 17.4 | -80 | -19 | -12 | d8-5S-HETE |
| LXA4 | 351.2 | 115.1 | 11.8 | -80 | -20 | -13 | d5-LXA4 |
| LXB4 | 351.2 | 221.1 | 11.4 | -80 | -20 | -13 | d5-LXA4 |
| LTB4 | 335.2 | 195.1 | 14 | -80 | -22 | -13 | d4-LTB4 |
| 20-COOH-LTB4 | 365.3 | 195.1 | 8.6 | -80 | -24 | -15 | d4-LTB4 |
| PGD2 | 351.3 | 233.1 | 11.1 | -80 | -16 | -15 | d4-PGE2 |
| PGE2 | 351.3 | 189.1 | 11 | -80 | -25 | -14 | d4-PGE2 |
| PGF2a | 353.3 | 193.1 | 11.7 | -80 | -34 | -11 | d4-PGE2 |
| TXB2 | 369.3 | 169.1 | 10.6 | -80 | -22 | -15 | d4-PGE2 |
| 12-HHT | 279.3 | 163.1 | 14.9 | -80 | -22 | -13 | d4-PGE2 |
| 5,6 EET | 319.2 | 191.1 | 18 | -80 | -21 | -12 | d8-5S-HETE |
| 8,9 EET | 319.2 | 127.1 | 18 | -80 | -21 | -12 | d8-5S-HETE |
| 11,12 EET | 319.1 | 167.1 | 17.1 | -80 | -21 | -12 | d8-5S-HETE |
| 14,15 EET | 319.2 | 219.1 | 17.1 | -80 | -18 | -12 | d8-5S-HETE |
| 5,6 DiHETrE | 337.2 | 145.1 | 17 | -80 | -25 | -12 | d8-5S-HETE |
| 8,9 DiHETrE | 337.2 | 127.1 | 16.5 | -80 | -30 | -12 | d8-5S-HETE |
| 11,12 DiHETrE | 337.2 | 167.1 | 16 | -80 | -25 | -12 | d8-5S-HETE |
| 14,15 DiHETrE | 337.2 | 207.1 | 15.5 | -80 | -25 | -12 | d8-5S-HETE |
| EPA | 301.3 | 257.1 | 17.9 | -100 | -16 | -18 | d8-5S-HETE |
| 5-HEPE | 317.2 | 115.1 | 17.6 | -80 | -18 | -12 | d8-5S-HETE |
| 12-HEPE | 317.2 | 179.1 | 16.8 | -80 | -19 | -12 | d8-5S-HETE |
| 15-HEPE | 317.2 | 219.1 | 16.4 | -80 | -18 | -12 | d8-5S-HETE |
| LXA5 | 349.2 | 115.1 | 10.6 | -80 | -20 | -13 | d5-LXA4 |
| LXB5 | 349.2 | 221.1 | 10.3 | -80 | -20 | -13 | d5-LXA4 |
| RvE2 | 333.3 | 253.1 | 12.5 | -80 | -20 | -12 | d5-RvD2 |
| RvE3 | 33.33 | 201.2 | 13.9 | -80 | -20 | -12 | d5-RvD2 |
| DHA | 327.3 | 283.1 | 18.5 | -100 | -16 | -18 | d8-5S-HETE |
| 7-HDHA | 343.2 | 141.1 | 17.5 | -80 | -18 | -15 | d8-5S-HETE |
| 14-HDHA | 343.2 | 205.1 | 17.5 | -80 | -17 | -14 | d8-5S-HETE |
| 17-HDHA | 343.2 | 245.1 | 17.5 | -80 | -17 | -14 | d8-5S-HETE |
| RvD1 & 2 | 375.2 | 141.1 | 11.5 | -80 | -21 | -13 | d5-RvD2 |
| RvD3 | 375.2 | 147.1 | 11.2 | -80 | -25 | -13 | d5-RvD2 |
| RvD4 | 375.2 | 101.1 | 12.7 | -80 | -22 | -16 | d5-RvD2 |
| RvD5 | 359.2 | 199.1 | 13.8 | -80 | -21 | -13 | d5-RvD2 |
| RvD6 | 359.2 | 101.1 | 14.6 | -80 | -22 | -16 | d5-RvD2 |
| NPD-1 | 359.2 | 153.1 | 13.6 | -80 | -21 | -9 | d5-RvD2 |
| Maresin 1 | 359.2 | 221.1 | 13.8 | -80 | -20 | -16 | d5-RvD2 |
| 22-COOH-MaR1 | 389.3 | 221.1 | 9.9 | -80 | -24 | -15 | d5-RvD2 |
| Maresin 2 | 359.1 | 221.2 | 14.9 | -80 | -20 | -12 | d5-RvD2 |
| DPA | 329.3 | 285.1 | 19 | -100 | -16 | -18 | d8-5S-HETE |
| DPA RvD1 | 377.2 | 215.1 | 11.6 | -80 | -26 | -13 | d5-RvD2 |
